# Primary Breast Malignancy in Children and Adolescents: A Population-Based Study

**DOI:** 10.1155/tbj/2919922

**Published:** 2024-12-05

**Authors:** Guorong He, Nan Shen, Lingling Zhao, Xian Liu, Caiyun Zhang

**Affiliations:** ^1^Department of Pediatrics Surgery, The Second Affiliated Hospital and Yuying Children's Hospital of Wenzhou Medical University, Wenzhou, China; ^2^Department of Pediatrics, Suqian Hospital Affiliated to Xuzhou Medical University, Suqian, Jiangsu 223800, China; ^3^Department of Pathology, The First Affiliated Hospital of Zhejiang Chinese Medical University (Zhejiang Provincial Hospital of Chinese Medicine), Hangzhou, Zhejiang, China; ^4^Department of Critical Care Medicine, Anji Branch of the First Affiliated Hospital of Zhejiang University, Anji County People's Hospital, Huzhou, Zhejiang, China; ^5^Department of Pediatric Intensive Care Unit, Hangzhou Children's Hospital, Hangzhou, Zhejiang, China

**Keywords:** breast cancer, children, infiltrating duct carcinoma, phyllodes tumor, survival

## Abstract

**Purpose:** Primary breast cancer in children and adolescents is extremely uncommon and presents with different characteristics from those found in adult women. We aimed to conduct a population-based cohort study to predict overall survival (OS) in pediatric patients with breast cancer.

**Methods:** Demographic and clinicopathological data on pediatric breast cancer patients were obtained from the Surveillance, Epidemiology, and End Results database (2000–2019). The survival rates were measured using the Kaplan–Meier method. Univariate survival analysis used the log-rank test, while multivariate analysis used Cox proportional hazards regression to identify factors influencing OS. Furthermore, we created a nomogram to predict OS in pediatric patients.

**Results:** A total of 115 pediatric patients were analyzed, with a median age at diagnosis of 18 years (range: 2–19 years). In terms of tumor grade, 27 (23.4%) patients had well or moderately differentiated tumors and 32 (27.8%) had poorly or undifferentiated tumors. The predominant histological type was phyllodes tumor, accounting for 36.5%, followed by infiltrating duct carcinoma at 31.3%, and other types at 32.2%. The SEER and M stages were substantial independent indicators of OS. A nomogram was created to predict OS in pediatric breast cancer patients.

**Conclusions:** Our findings confirmed that the SEER stage and M stage were the most critical predictors of OS in pediatric patients with breast cancer. By focusing on this rare demographic, our study fills an important gap in the literature, as there are few comprehensive studies available that explore a prognosis in pediatric breast cancer.

## 1. Introduction

Breast cancer in females under 20 years of age is reported to occur at a rate of 2–8 per 100,000 [1, 2]. Among children and adolescents, the predominant malignant primary breast tumors are malignant phyllodes tumors, followed by infiltrating duct carcinomas [3, 4]. Typically, carcinomas are observed slightly more frequently than sarcomas [4]. The literature suggests that primary breast tumors in pediatric patients generally exhibit a poorer prognosis compared to those in adults [5–7], making it a continuous focus of interest in pediatric surgical studies. In addition, the research also suggests that phyllodes tumors in pediatric patients exhibit distinct characteristics compared to those in adults [8].

In our study, we analyzed the largest cohort sourced from the SEER database spanning from 2000 to 2019, hypothesizing that our results might provide a factual perspective on the features and prognosis of breast cancer in children and adolescents. Furthermore, we assessed the morbidity rates and survival outcomes across various pathological types of breast cancers in this group.

## 2. Methods

### 2.1. Study Population

Between 2000 and 2019, we utilized the SEER database to catalog all cases of pediatric breast cancer. We identified pediatric and adolescent patients (aged ≤ 19 years) diagnosed with breast cancer using the International Classification of Diseases for Oncology, Third Edition (ICD-O-3), site codes C50.1 through C50.9. The classification of breast cancers was based on ICD-O-3 morphological codes, covering subtypes such as phyllodes tumor (code 9020), infiltrating duct carcinoma (code 8500), hemangiosarcoma (code 9120), and secretory carcinoma of the breast (code 8502) (Table 1). Our dataset was carefully checked to ensure it contained no duplicates, and since the data were anonymized, neither informed consent nor ethical review was necessary.

We extracted demographic and clinical data from the database, including patient race (categorized as White or others), tumor grade (ranging from well and moderate differential to poorly and undifferentiated), and TNM staging (using the sixth edition of the Union for International Cancer Control/American Joint Committee on Cancer system). Cancer stages were determined according to SEER's historic staging criteria, which classify the disease as localized, regional, or distant. The study participants were divided into two age groups: 0–15 years and 16–19 years, with 0–15 years typically encompassing prepuberty and early puberty, while 16–19 years reflects later stages of puberty and near-adult physiology. Information on their treatments such as surgery, chemotherapy, and radiotherapy was also included. The primary endpoint of our analysis was overall survival (OS), defined from the point of diagnosis to the last follow-up or death.

### 2.2. Statistical Analysis

All statistical analyses in this study were performed using SPSS software (version 22.0, SPSS Inc., Chicago, Illinois, United States of America). Descriptive statistics were computed for each variable. We generated Kaplan–Meier survival curves for 1-year, 3-year, and 5-year OS intervals and evaluated them using the log-rank test. To explore the independent predictors of outcomes, we employed multivariate Cox proportional hazards regression models, reporting hazard ratios (HRs). In addition, we created a nomogram based on the multivariate Cox regression model to predict OS. To assign a point value to each category of each variable, we draw a vertical line to the point's axis. Then, we add up all of the point values for the variables to get the total point for this patient. Lastly, we drop a vertical line from the total points' axis to the 3- and 5-year survival probability axes, respectively. Statistical significance was determined using a two-tailed *p* value threshold of 0.05.

## 3. Results

### 3.1. Patient Characteristics

Between 2000 and 2019, 115 pediatric and adolescent patients were identified with breast cancer diagnoses. The median diagnosis age was 18 years, ranging from 2 to 19 years. Of these, 29 patients (25.3%) were 15 years old or younger, while 86 patients (74.7%) were older than 15 years. The cohort included only one male patient. Regarding tumor grades, 27 patients (23.4%) had well or moderately differentiated tumors and 32 patients (27.8%) had poorly differentiated or undifferentiated tumors. Phyllodes tumors were the most common histological type, representing 36.5% of cases, followed by infiltrating duct carcinoma at 31.3%, and other types comprising 32.2%. Distant metastasis was found in 11.3% of the patients, while 70.4% had localized disease and 18.3% had regional disease. The TNM staging distribution was as follows: T1 (44.3%), T2 (40%), T3 (10.4%), and T4 (5.3%); N0 (69.5%) and N1–N3 (30.5%); and M0 (86.9%) and M1 (13.1%). Most patients (89.5%) underwent surgical treatment, with 43.5% receiving chemotherapy and 29.5% undergoing radiotherapy.  Table 2 presents the validated subtypes of breast cancer in the 115 patients. Those with phyllodes tumors typically presented with localized disease, while other tumor types were more frequently associated with lymph node metastases (*p* < 0.001).

### 3.2. Survival and Prognosis Analysis

**Figure 1:**
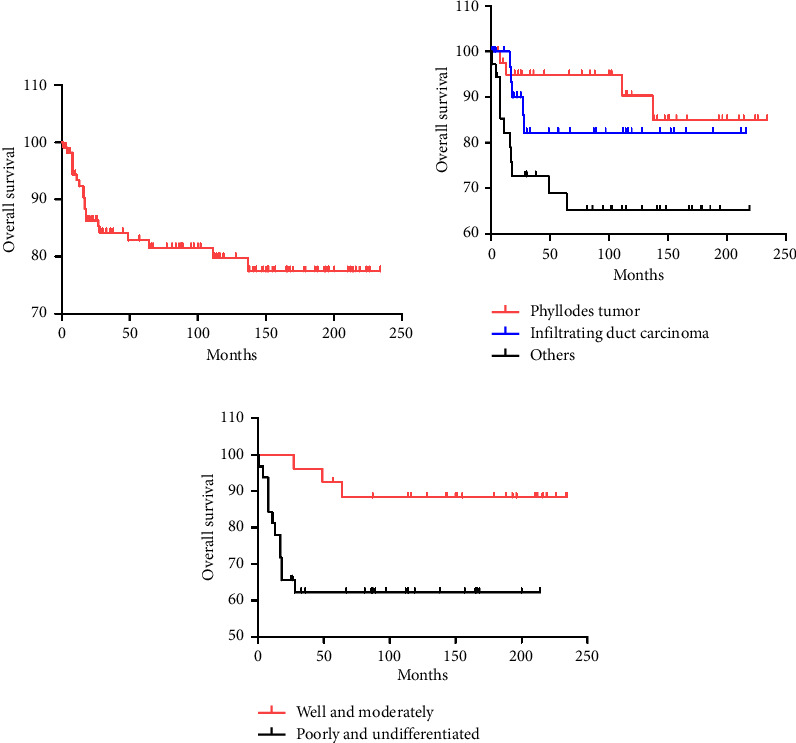
Kaplan–Meier survival curves for the entire cohort and by a subgroup: (a) by overall; (b) by histology; (c) by grade.

**Figure 2:**
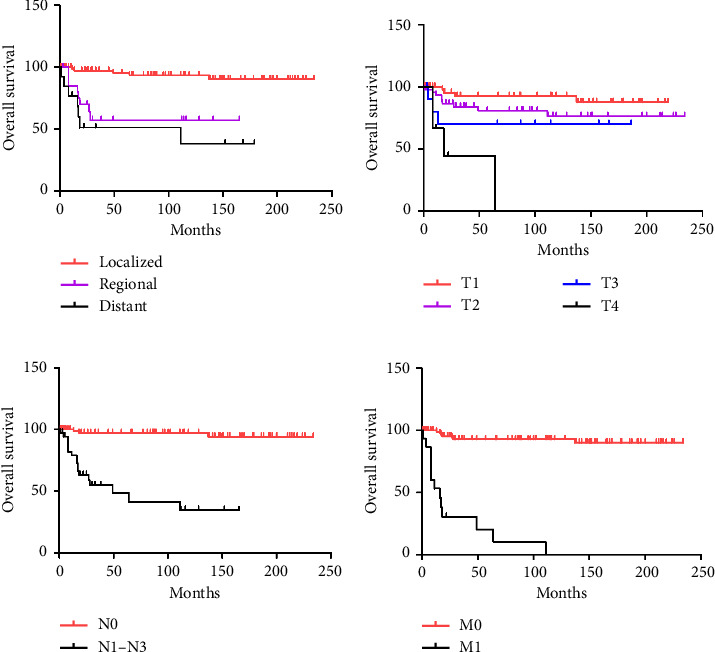
Kaplan–Meier survival curves for children and adolescents with breast cancer, when stratified by the SEER stage and TNM stage: (a) by SEER stage; (b) by T stage; (c) by N stage; (d) by M stage.

All patients exhibited 1-year, 3-year, and 5-year OS rates of 93.4%, 84.1%, and 82.9%, respectively (Table 3 and Figure 1(a)). There were no significant differences in OS based on race, or age at diagnosis (*p*=0.724, *p*=0.260, respectively) (Table 4). However, patients with phyllodes tumors showed significantly better OS outcomes (*p*=0.02) (Figure 1(b)). In addition, patients with well or moderately differentiated tumors had superior survival rates compared to those with poorly or undifferentiated tumors (5-year OS, well or moderately grade 92.6% versus 62.2% for poorly or undifferentiated grade, *p*=0.016) (Figure 2(c)). Moreover, patients with localized disease had significantly better survival compared to those with regional or distant disease (*p* < 0.001) (Figure 2(a)). Specifically, T stage, N stage, and M stage emerged as independent prognostic factors for OS (Figures 2(b), 2(c), and 2(d)). In terms of treatment, patients who did not undergo chemotherapy or radiotherapy had better survival outcomes (*p* < 0.01).

**Figure 3:**
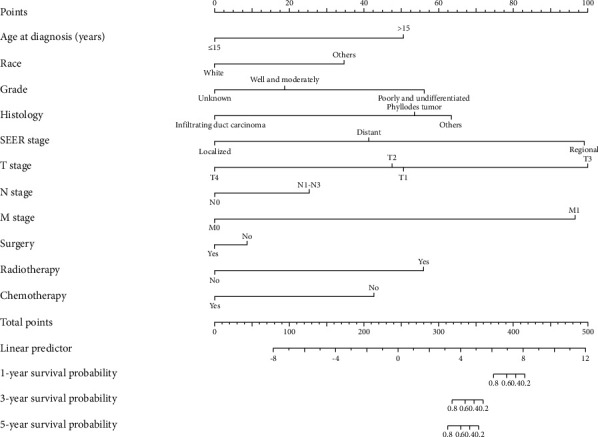
Nomogram for predicting 1-, 3-, and 5-year overall survival probability.

Table 4 summarizes the results from the Cox regression multivariate analysis. The analysis highlighted the SEER stage and M stage as significant independent predictors of OS. Specifically, patients in the M1 stage had a significantly higher risk of death (HR 73.7, 95% confidence interval [CI] 6.1–889; *p* < 0.001) compared to those in the M0 stage. A nomogram was constructed to predict OS in pediatric breast cancer patients, illustrating an increased probability of death in patients with regional or M1 stage disease (Figure 3).

## 4. Discussion

Breast cancer in children and adolescents is exceedingly rare, with most cases occurring in adults over the age of 40. However, it is important to discuss its occurrence in younger populations due to its significant impact when it does occur [9]. Utilizing the SEER database, this study conducted a comprehensive population-based review of pediatric breast cancer from 2000 to 2019, identifying phyllodes tumors and infiltrating duct carcinoma as the most prevalent histological types. Both univariate and multivariate analyses highlighted the SEER stage and M stage as crucial determinants of OS. We also developed a nomogram to predict survival probabilities at 1, 3, and 5 years, offering a valuable prognostic tool for pediatric breast cancer.

The majority of diagnosed patients are females aged 15–19 years, accounting for 74.7% of cases, with a median age of 18 years. Tumors are predominantly phyllodes tumor (36.5%), followed by infiltrating duct carcinoma (31.3%), and other types (32.2%). Phyllodes tumors are rare fibroepithelial neoplasms that are characterized by a biphasic pattern of stromal and epithelial components. Infiltrating duct carcinoma is the most common type of breast cancer, which originates in the milk ducts and invades the surrounding breast tissue. The prognosis of breast tumor is influenced by several factors such as tumor size, grade, lymph node involvement, hormone receptor status, and HER2 overexpression. The majority of tumors are diagnosed at an early stage, with localized forms accounting for 70.4% of cases. Despite the predominance of early-stage diagnosis, pediatric breast cancer exhibits a more aggressive progression than in adults [10]. Our study found a 5-year survival rate difference by histology and stage but not by race or age at diagnosis. Specifically, the 5-year survival rates for phyllodes tumor and infiltrating duct carcinoma were 94.9% and 82.2%, respectively, compared to 69.0% for other histological types. The study also confirmed high-grade and advanced-stage diseases were associated with poorer outcomes, consistent with previous studies [11, 12]. In our cohort, while the advanced SEER stage was linked to increased mortality, T and N stages did not significantly impact OS. Patients with distant metastases faced a considerably higher risk of death.

The treatment of pediatric breast cancer is rare but requires careful consideration due to its unique challenges and implications for a growing child. Surgery is often a primary treatment option for breast cancer in pediatric patients [13]. This may involve a lumpectomy (removal of the tumor and some surrounding tissue) or mastectomy (removal of the entire breast), depending on the extent of the cancer. In our research, a large proportion of patients (89.5%) underwent surgical intervention. According to guidelines, chemotherapy is generally not advised, and radiotherapy is suggested as a category 2B treatment only after a tumor recurs following initial surgical resection [14]. Over the past two decades, the application of radiotherapy for malignant phyllodes tumors in the breast has increased [15, 16], yet there is no substantial evidence that it enhances OS or tumor-specific survival for these patients [17]. Given the potential for inducing secondary malignant tumors, the use of radiotherapy in children requires careful consideration [18]. Our data suggest that both chemotherapy and radiotherapy were linked to worse OS outcomes, possibly because these treatments are more frequently used in palliative care settings or in patients with advanced stages of breast cancer.

This study is subject to several limitations. The absence of family history data limits a thorough evaluation of a patient's breast cancer risk. In addition, while data on surgical interventions were collected, detailed information on the extent of surgical resection was not available. The lack of a validation cohort, due to the small sample size, may also compromise the robustness of our predictive model. Furthermore, the predominance of White patients in the SEER database may limit the generalizability of our findings across different racial groups, necessitating further studies to validate these results.

In conclusion, pediatric breast cancer, though rare, often exhibits more aggressive features compared to adult cases, including higher grade tumors, increased likelihood of metastasis, and a more variable prognosis based on tumor subtype. The SEER stage and M stage were identified as the primary predictors of survival. Ongoing research and clinical innovation are crucial to enhance early detection and develop treatment approaches specifically designed for the unique challenges faced by pediatric breast cancer patients.

## Figures and Tables

**Table 1:** Histologic subtypes and frequency encountered.

Histology	Frequency, *n* (%)
Phyllodes tumor	42 (36.5)
Infiltrating duct carcinoma	36 (31.3)
Others	37 (32.2)
Hemangiosarcoma	7
Neoplasm, malignant	5
Secretory carcinoma of the breast	5
Alveolar rhabdomyosarcoma	3
Giant cell sarcoma	3
Carcinosarcoma	2
Ewing sarcoma	2
Carcinoma, NOS	2
Adenoid cystic carcinoma	1
Mucinous adenocarcinoma	1
Inflammatory carcinoma	1
Metaplastic carcinoma	1
Dermatofibrosarcoma, NOS	1
Leiomyosarcoma, NOS	1
Rhabdomyosarcoma, NOS	1
Sarcoma, NOS	1

**Table 2:** Basic characteristics of patients stratified by tumor subtypes.

Characteristics	Phyllodes tumor	Infiltrating duct carcinoma	Others	*p* value
*n*	42	36	37	
Age at diagnosis (years), *n* (%)				0.001
>15	31 (27%)	34 (29.6%)	21 (18.3%)	
≤15	11 (9.6%)	2 (1.7%)	16 (13.9%)	
Race, *n* (%)				0.012
White	22 (19.1%)	23 (20%)	31 (27%)	
Others	20 (17.4%)	13 (11.3%)	6 (5.2%)	
Grade, *n* (%)				0.196
Well and moderately differentiated	8 (7%)	12 (10.4%)	7 (6.1%)	
Poorly and undifferentiated	9 (7.8%)	12 (10.4%)	11 (9.6%)	
Unknown	25 (21.7%)	12 (10.4%)	19 (16.5%)	
SEER stage, *n* (%)				< 0.001
Localized	40 (34.8%)	17 (14.8%)	24 (20.9%)	
Regional	1 (0.9%)	14 (12.2%)	6 (5.2%)	
Distant	1 (0.9%)	5 (4.3%)	7 (6.1%)	
T stage, *n* (%)				0.198
T1	13 (11.3%)	18 (15.7%)	20 (17.4%)	
T2	22 (19.1%)	14 (12.2%)	10 (8.7%)	
T3	6 (5.2%)	2 (1.7%)	4 (3.5%)	
T4	1 (0.9%)	2 (1.7%)	3 (2.6%)	
N stage, *n* (%)				< 0.001
N0	39 (33.9%)	19 (16.5%)	22 (19.1%)	
N1–N3	3 (2.6%)	17 (14.8%)	15 (13%)	
M stage, *n* (%)				0.008
M0	40 (34.8%)	33 (28.7%)	27 (23.5%)	
M1	2 (1.7%)	3 (2.6%)	10 (8.7%)	
Surgery, *n* (%)				< 0.001
Yes	42 (36.5%)	34 (29.6%)	27 (23.5%)	
No	0 (0%)	2 (1.7%)	10 (8.7%)	
Radiotherapy, *n* (%)				< 0.001
No	39 (33.9%)	21 (18.3%)	21 (18.3%)	
Yes	3 (2.6%)	15 (13%)	16 (13.9%)	
Chemotherapy, *n* (%)				< 0.001
No	40 (34.8%)	9 (7.8%)	16 (13.9%)	
Yes	2 (1.7%)	27 (23.5%)	21 (18.3%)	

**Table 3:** 1-, 3-, and 5-year survival for entire cohort and by subgroup.

Feature	1-Year OS (%)	3-Year OS (%)	5-Year OS (%)
Overall	93.4	84.1	82.9
Race			
White	91.1	86.4	84.4
Others	97.4	80.3	80.3
Age at diagnosis (years)			
≤15	96.0	92.0	92.0
>15	92.5	81.5	79.8
Histology			
Phyllodes tumor	97.5	94.9	94.9
Infiltrating duct carcinoma	96.7	82.2	82.2
Others	82.1	72.7	69.0
Grade			
Well and moderately differentiated	96.3	92.6	92.6
Poorly and undifferentiated	81.3	62.2	62.2
Unknown	97.8	92.9	92.9
SEER stage			
Localized	98.5	97.1	95.3
Regional	85.0	57.3	57.3
Distant	76.9	51.3	51.3
T stage			
T1	100	92.4	92.4
T2	93.3	84.0	81.0
T3	80.0	70.0	70.0
T4	66.7	44.4	44.4
N stage			
N0	100	97.1	97.1
N1–N3	79.0	54.8	48.7
M stage			
M0	100	92.8	92.8
M1	53.3	30.5	20.3
Surgery			
Yes	94.7	85.5	84.0
No	81.8	72.7	72.7
Radiotherapy			
Yes	86.5	68.7	64.1
No	96.1	90.4	90.4
Chemotherapy			
Yes	96.7	93.1	93.1
No	88.8	71.6	68.5

**Table 4:** Survival analyses of overall survival for pediatric breast cancer.

Characteristics	Univariate analysis		Multivariate analysis	
	Hazard ratio (95% CI)	*p* value	Hazard ratio (95% CI)	*p* value
Age at diagnosis (years)				
>15	Reference			
≤15	0.494 (0.145–1.685)	0.260		
Race				
White	Reference			
Others	0.851 (0.348–2.083)	0.724		
Grade				
Well and moderately differentiated	Reference		Reference	
Poorly and undifferentiated	4.803 (1.347–17.128)	**0.016**	4.000 (0.603–26.554)	0.151
Unknown	1.255 (0.298–5.280)	0.757	0.407 (0.039–4.229)	0.452
Histology				
Phyllodes tumor	Reference		Reference	
Infiltrating duct carcinoma	1.696 (0.455–6.328)	0.432	0.334 (0.041–2.687)	0.302
Others	3.878 (1.234–12.186)	**0.020**	1.552 (0.315–7.648)	0.589
SEER stage				
Localized	Reference		Reference	
Regional	7.832 (2.536–24.184)	**< 0.001**	27.798 (2.438–316.944)	**0.007**
Distant	12.093 (3.816–38.327)	**< 0.001**	3.870 (0.441–33.946)	0.222
T stage				
T1	Reference		Reference	
T2	2.461 (0.757–8.005)	0.134	0.405 (0.064–2.576)	0.338
T3	3.960 (0.886–17.710)	0.072	5.411 (0.423–69.235)	0.194
T4	15.318 (3.706–63.309)	**< 0.001**	0.146 (0.014–1.533)	0.109
N stage				
N0	Reference		Reference	
N1–N3	21.885 (6.212–77.100)	**< 0.001**	3.874 (0.310–48.476)	0.294
M stage				
M0	Reference		Reference	
M1	31.007 (11.419–84.199)	**< 0.001**	73.739 (6.111–889.705)	**< 0.001**
Surgery				
Yes	Reference			
No	1.557 (0.456–5.316)	0.480		
Radiotherapy				
No	Reference		Reference	
Yes	3.702 (1.525–8.984)	**0.004**	3.366 (0.625–18.131)	0.158
Chemotherapy				
No	Reference		Reference	
Yes	3.943 (1.507–10.313)	**0.005**	0.339 (0.048–2.410)	0.280

*Note:* Bold values indicate < 0.05.

## Data Availability

The dataset used and analyzed during the current study are available from the corresponding author on reasonable request.
